# Characterizing the impact of *MLL* fusion variants and fusion partners on leukemia plasticity using a human CRISPR-engineered *MLL*-rearranged leukemia model

**DOI:** 10.1016/j.neo.2026.101308

**Published:** 2026-04-18

**Authors:** Pia Radszuweit, Rahel Fitzel, Sarah Bruestl, Thomas Hentrich, Fulya Korkmaz, Barbara Mankel, Irene González-Menéndez, Saskia Rudat, Rolf Marschalek, Estelle Erkner, Hildegard Keppeler, Rebekka Schairer, Luise Luib, Markus Mezger, Leticia Quintanilla-Martinez, Julia Schulze-Hentrich, Claudia Lengerke, Dominik Schneidawind, Corina Schneidawind

**Affiliations:** a Department of Medical Oncology and Hematology, University Hospital Zurich, Zurich, Switzerland; b Department of Internal Medicine II, University Hospital Tübingen, Tübingen, Germany; c Department of Genetics/Epigenetics, Saarland University, Saarbrücken, Germany; d Institute of Pathology and Neuropathology and Comprehensive Cancer Center, University Hospital Tübingen, Tübingen, Germany; e Core Facility Histology, Faculty of Medicine, University of Tübingen, Tübingen, Germany; f Institute of Pharmaceutical Biology, Goethe-University Frankfurt, Frankfurt/Main, Germany; g Department of General Paediatrics, Haematology/Oncology, University Children's Hospital Tübingen, Tübingen, Germany; h Cluster of Excellence iFIT (EXC 2180) “Image-Guided and Functionally Instructed Tumor Therapies”, Eberhard Karls University, Tübingen, Germany

**Keywords:** *KMT2A*-rearrangements, Leukemia modeling, CRISPR/Cas9-engineered models, *in vivo* leukemia modeling, Hematopoetic stem cells, Acute leukemia

## Abstract

Acute leukemias involving *KMT2A* (*MLL*) rearrangements are aggressive hematologic malignancies associated with a poor prognosis, especially in infants. The majority of *MLL* breakpoints are located within the breakpoint cluster region spanning exons 8-14, with *AFF1* (*AF4*) and *MLLT3* (*AF9*) being the most frequent fusion partners. To study the contribution of different fusion partners and breakpoint locations to leukemogenesis, we created a human CRISPR/Cas9-based model. We introduced *MLL::AF4* or *MLL::AF9* fusions with *MLL* breakpoints in intron 9 or 11, respectively, into human cord blood-derived CD34^+^ cells from the same donor. Compared to healthy control cells, all *MLL*-rearranged cells showed increased proliferation and stemness, as well as an altered immunophenotype characterized by the upregulation of leukemic markers. Transcriptomic profiling revealed breakpoint- and partner-specific gene expression patterns that influence the characteristics of the disease. Notably, even after prolonged *in vitro* culture MLL(intron 9)::AF9 cells displayed robust colony formation in semisolid media and engrafted robustly in NOD *scid* gamma mice. The cells still exhibited high lineage plasticity, switching from a myeloid to a B-lymphoid identity *in vivo*. In conclusion, this model enables the mechanistic dissection of *MLL* fusion variants *in vitro* and *in vivo*, providing a foundation for developing targeted therapies for *MLL*-rearranged leukemias.

## Highlights

• CRISPR-engineered HSPCs model KMT2A-rearrangement heterogeneity *in vitro* & *in vivo*.
• Transcriptome of KMT2A-rearranged cells matches KMT2A hallmark signatures.
• KMT2A(int.9)::MLLT3 cells engraft superiorly presumably via M2-like macrophages.
• Model captures lineage plasticity via myeloid-to-lymphoid switching *in vivo*.
• Future platform for drug testing and KMT2A-tailored precision medicine.

## Introduction

Rearrangements of the Histone-lysine N-methyltransferase 2A gene (*KMT2A*, also known as *MLL*), located on chromosome 11q23, occur in 5-10% of acute leukemias, with particularly high prevalence in infants and children [1,2]. Common *MLL* fusion partners include *AF4* (*AFF1,* ALF Transcription Elongation Factor 1), *AF9* (*MLLT3*, MLLT3 Super Elongation Complex Subunit), *MLLT1, MLLT10, ELL, AFDN*, and *EPS15* [3]. *MLL::AF9* predominates in acute myeloid leukemia (AML), while *MLL::AF4* is more frequent in acute lymphoid leukemia (ALL) [4].

The MLL protein is cleaved by Taspase-1 into the fragments MLL-N (∼320 kDa) and MLL-C (∼180 kDa), which reassemble with other binding proteins into a multiprotein complex regulating histone modification and gene expression [5]. MLL-N contains the Menin-binding domain, where MLL-bound Menin recruits LEDGF to recognize histone 3 lysine 36 (H3K36) methylation marks, facilitating MLL recruitment to active chromatin [6,7]. The middle portion of MLL contains four plant homeodomains (PHD1-4) mediating transcriptional repression (via the BMI1 complex) [8] or activation (via H3K4me3 recognition) [9]. Chromosomal translocations at 11q23 lead to the functional disruption of the MLL protein complex, abolishing repressor recruitment and causing constitutive activation [10]. While the MLL::X fusion mainly drives aberrant transcriptional programs, the reciprocal X::MLL fusion promotes leukemic transformation by chromatin-remodeling activity [11,12].

In 94% of *MLL*-rearranged (*MLL*r) leukemia patients, the breakpoint within the *MLL* gene occurs in the major breakpoint cluster region (especially introns 9-11) with distribution varying by age and leukemia subtype [4,13]. Breakpoints in intron 11 predominate in pediatric ALL, while intron 9 breakpoints are more common in AML and older patients. Previous findings suggest that intron 11 breakpoints associate with worse prognosis compared to intron 9^13^. One explanation involves the PHD subdomain disruption^14^: Intron 11 breakpoints disrupt PHD1/2 folding, compromising dimerization, diminishing repressor recruitment, and enhancing the activator function [14]. In contrast, intron 9 breakpoints preserve an intact PHD structure.

*MLL*r leukemias respond poorly to standard chemotherapy regimens and clinical outcomes remain poor. We developed novel human *MLL*r models harboring *MLL::AF4* and *MLL::AF9* with breakpoint in intron 9 and compared them to our established models with breakpoint in intron 11^15^. All *MLL* fusions altered the immunophenotype, and both fusion partner and breakpoint location influenced the transcriptional landscape. MLL(intron 9)::AF9 cells engrafted robustly in xenograft experiments, allowing for a thorough characterization of the cells *in vivo* and a comparison with *in vitro*-cultured cells.

## Methods

### Generation and cultivation of *MLL*r cells

Human umbilical cord blood (huCB) was obtained from the Center for Women’s Health of the University Hospital Tübingen (IRB approvals: 751/2015BO2, 461/2022BO2). Written consent was obtained from all patients in compliance with the Declaration of Helsinki. CRISPR/Cas9-mediated induction of *MLL::AF4* and *MLL::AF9* rearrangements in huCB-derived CD34^+^ hematopoietic stem and progenitor cells (HSPCs) and the culture conditions for *MLL*r cells were described previously [15,16] (sgRNA target sequences in Supplementary Table S1).

### Genomic PCR for translocation and mutation detection

For translocation detection the PrimeSTAR Max DNA Polymerase Mix (Takara, Cat# R045A) was used. For *KRAS* mutation detection AmpliTaq Gold 360 Mastermix (Thermo Fisher Scientific, Cat# 4398876) and M13-tailed primer pairs were used (Thermo Fisher Scientific, Assay IDs: Hs00679698_CE, Hs00532900_CE). All primers listed in Supplementary Table S2.

### ddPCR

To perform digital droplet PCR, the Bio-Rad QX200 system and 2x ddPCR EvaGreen Supermix (BioRad, 186-4033) were used. The reaction was set up according to manufacturer’s instructions using 220 ng of gDNA. Albumin (*ALB*) was used as a reference gene. From the raw data (given in copies/L), the mean values of duplicates were calculated for the reference gene and the respective fusion gene. The ALB values were normalized to the wild-type (wt) sample to account for technical variability. Subsequently, the mean values of the fusion gene were divided by the normalized ALB values to normalize the amplicons of the fusion gene to the total DNA content. The value of the wt sample was subtracted from the result, and the remaining values were scaled relative to the 100% control (100% *MLL*r cells) to determine the percentage of *MLL*r cells. Primers are listed in Table S3.

### FISH and karyotyping

Fluorescence *in situ* hybridization was performed as previously described [17] using the ZytoLight SPEC *KMT2A* Dual Color Break Apart Probe (ZytoVision GmbH, Cat# Z-2193-50).

Karyotyping was performed by the Department of Medical Genetics and Applied Genomics at the University Hospital of Tübingen following a standard protocol.

### Cytospins and Pappenheim staining

2 × 10^5^ cells were harvested and centrifuged in a cytocentrifuge (Cytospin 4, Thermo Fisher Scientific). The cells were first stained in Giemsa solution, followed by staining with May-Gruenwald solution. Per slide, 200 cells were examined to determine lineage specification.

### Flow cytometry and cell sorting

After Fc receptor blocking (Miltenyi Biotec, Cat# 130-059-901) the cells were stained with the antibody mix. To analyze and sort bone marrow cells of mice, Fc receptor blocking was performed using IgG from human Serum (Sigma-Aldrich, Cat# I4506) and Mouse BD Fc Block (BD Biosciences, Cat# 553141). Antibodies are listed in the supplementary methods. Flow cytometry measurements and cell sorting were performed at the Flow Cytometry Core Facility Tübingen using a BD LSRFortessa and a BD FACS Aria IIIu (BD Biosciences), respectively. Data were analyzed with FlowJo v10.10.

### Cytarabine treatment and Annexin V/PI staining

After 72 h of cytarabine treatment, cells were stained for Annexin V/PI (Miltenyi Biotec, Cat# 130-092-052). Measurements were performed on a BD LSRFortessa (BD Biosciences) and analyzed with FlowJo v10.10.

### CFU assay

To determine the self-renewing capacity of the cells, 5000 cells were washed and resuspended in 100 µl PBS. The cell suspension was added to 4 ml prewarmed methylcellulose (Biotechne, HSC003) and 3 × 1 ml were plated as technical triplicates into a 10/35 mm cell culture dish. After 14 days, the colonies were counted.

### MACE-seq

Wt and *MLL*r cells were harvested 71 to 78 days after translocation induction. Library preparation and sequencing was performed at GenXPro GmbH (Frankfurt/Main, Germany) using the MACE-Seq Kit (GenXPro GmbH). Fragmented DNA-depleted RNA was reverse transcribed, followed by template switching combined with the incorporation of the TrueQuant UMI. The cDNA fragments were PCR amplified and purified. Sequencing was performed on an Illumina NextSeq instrument.

### RNA-seq

RNA isolation, library preparation and RNA sequencing was performed at the Department of Genetics and Epigenetics, Saarland University (Saarbrücken, Germany). A modified SmartSeq 2 protocol was applied. The final libraries were prepared by using the Nextera DNA Library Preparation Kit (Illumina, Cat# FC‐131‐1024). The libraries were sequenced on the AVITI platform (Element Biosciences).

### Histology of mouse organs

Histology of mouse organs was performed at the Histology Core Facility section Mouse Pathology of the University Hospital Tübingen. For histology 3-5 µm-thick sections were cut from the femur and stained with hematoxylin and eosin (H&E). All slides were scanned with the Ventana DP200 (Roche) and processed with the Image Viewer MFC Application. Final image preparation was performed with Adobe Photoshop CS6.

### Xenograft experiments

NOD.Cg-*Prkdc^scid^ IL2rg^tmWjl^*/Sz mice (strain code 614) were purchased from Charles River Laboratories (Sulzfeld, Germany) and were bred in-house. Experiments were performed following approval by the local authorities. Six- to ten-week-old female or male NSG mice were preconditioned by sub-lethal irradiation and 24 h later 3 × 10^6^ *MLL*r cells were injected into the tail vein. *MLL*r cells were cultured for 94 days (Donor 1) or 88 days (Donor 2) after translocation induction. At signs of sickness or after a maximum follow-up time of one year, the mice were sacrificed.

### Statistical analysis

GraphPad Prism 10 was used to perform statistical analysis as specified in the figure descriptions. Data are presented as mean with standard deviation (SD). Statistical significances were defined as follows: not significant (ns) p > 0.05; *p ≤ 0.05; **p ≤ 0.01; ***p ≤ 0.001; **** p ≤ 0.0001. MACE- and RNA-sequencing data processing are described in the supplementary materials.

## Results

### Inducing *MLL* rearrangements using CRISPR/Cas9 leads to increased proliferation and outgrowth of *MLL*r cells

**Fig. 1:**
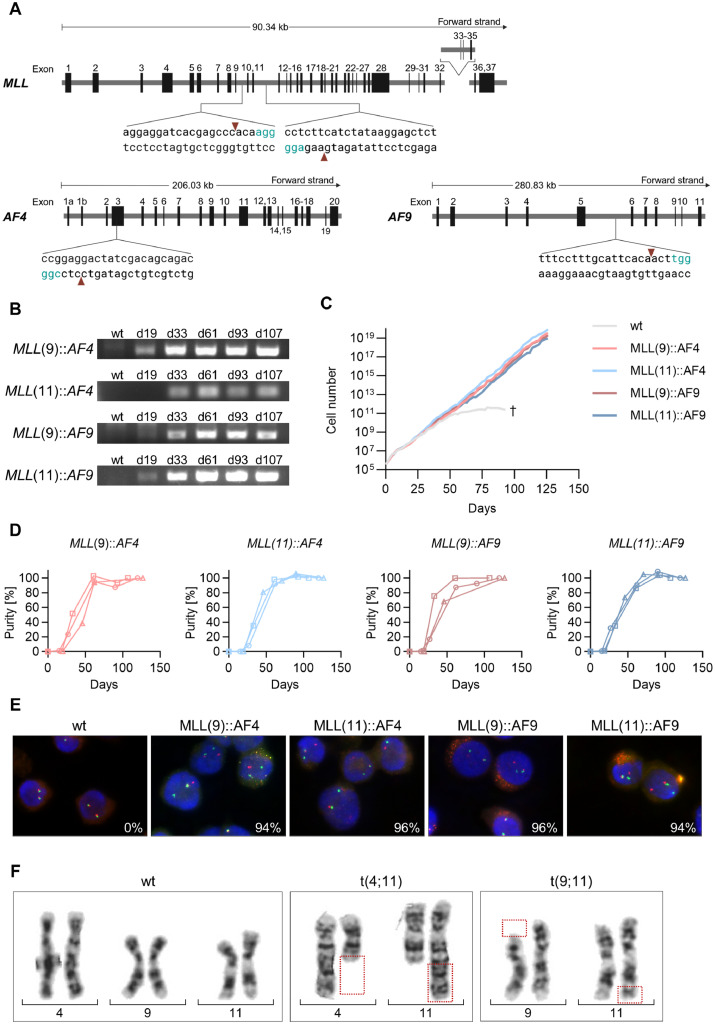
**After inducing *MLL* rearrangements, *MLL-*rearranged (*MLL*r) cells outgrow wild-type (wt) cells within 40-70 days. A** Schematic representation of sgRNA cutting sites in *MLL*, the *AF4*, and *AF9* gene. Figure created with Biorender.com. **B** Genomic PCR was used to detect the fusion of *MLL* intron 9 or 11 with *AF4* or *AF9* in CRISPR/Cas9-modified cells 19 to 107 days (d) after translocation induction. Results of one representative donor are shown. **C** Representative growth curves of cells of one donor after translocation induction. Cell growth was frequently documented by cell counting with trypan blue. **D** Digital droplet PCR was performed on genomic DNA to quantify the purity of *MLL*r cells. The presence of the respective *MLL* fusion gene and ALB was measured*.* Raw data (copies/L) were normalized to ALB values and scaled relative to a 100% *MLL*r control. Each line represents one donor. **E** Fluorescence *in situ* hybridization analysis of wt and *MLL*r cells using an *MLL* Dual Color break apart probe. One orange signal, and one separate green signal indicates that one 11q23.3 locus is involved in a translocation. Representative pictures of one donor, and the percentage of *MLL*r cells are shown. **F** Representative G-banded karyotypes of wt and *MLL*r cells of one donor. Chromosomes 4, 9 and 11 are shown. t(4;11) includes cells with a fusion of *MLL* and *AF4*. t(9;11) includes cells with a fusion of *MLL* and *AF9*.

To induce *MLL* rearrangements, CD34^+^ HSPCs were isolated from huCB and nucleofected with Cas9 protein, an sgRNA targeting *MLL* intron 9 or 11 and an sgRNA targeting *AF4* or *AF9* (Fig. 1A). Cas9 alone was used for control cells (wild-type, wt) of the same donor. *MLL* fusion products and reciprocal fusions were detected by genomic PCR (Fig. 1B, Supplementary Figure S1B) and the expected fusion sequences were validated by Sanger sequencing (Supplementary Figure S1A). The signal intensity of fusion products increased over time, indicating growing proportions of translocated cells. Around day 50 after translocation induction, the proliferation rate of control cells decreased, while the *MLL*r cells kept their proliferation rate at the same level (Fig. 1C). Digital droplet PCR confirmed that the proliferative advantage of *MLL*r cells led to a 100% pure culture within 40 to 70 days (Fig. 1D). *MLL* rearrangements were validated by fluorescence *in situ* hybridization and karyotyping (Fig. 1E, F). The control cells exhibited a normal karyotype, while cells with *MLL::AF4* fusion carried the t(4;11) (q21;q23) translocation, and MLL::AF9 cells displayed a karyotype with t(9;11) (p22;q23) (Fig. 1F, Supplementary Figure S2). *MLL::AF4* and *MLL::AF9* fusion transcript expression was confirmed by RT-PCR (Supplementary Figure S3).

### CRISPR/Cas9-generated *MLL*r cells are characterized by high blast counts and immaturity markers reflecting differentiation arrest

**Fig. 2:**
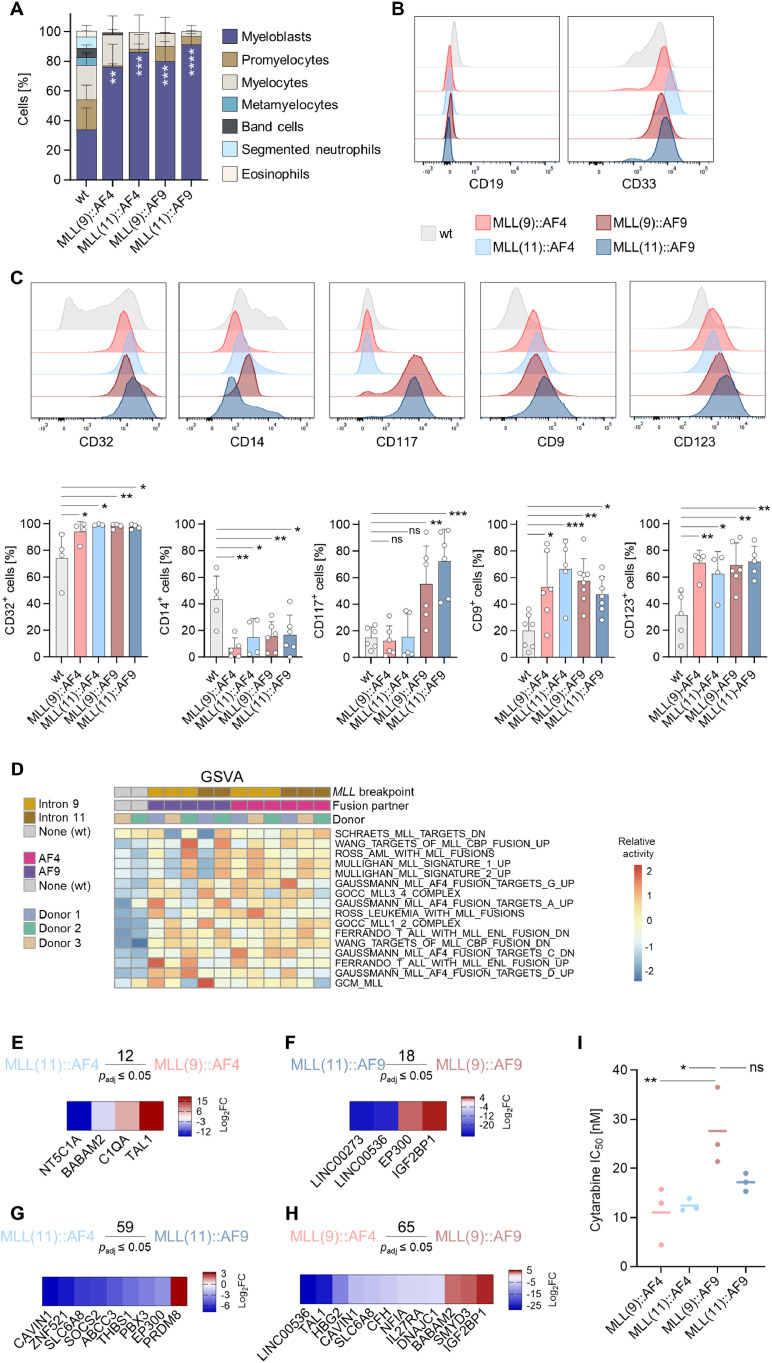
***MLL-*rearranged (*MLL*r) cells display AML-like morphology and phenotype, while their transcriptome matches *MLL*r hallmark signatures shaped by *MLL* breakpoint and fusion partner. A** Quantification of each cell type in cytospins of *MLL*r and wt cells. Cells were stained by Pappenheim staining. Results of three independent donors are shown. One-way ANOVA with Dunnett's multiple comparisons test was performed to compare the percentage of myeloblasts to the wt control. **B** Flow cytometric analysis of wt and *MLL*r cells for CD19 and CD33 surface expression. Representative histograms are shown. **C** Flow cytometric analysis of *MLL*r and wt cells. The cells were analyzed for the surface expression of CD32, CD14, CD117, CD9 and CD123. Representative histograms (upper panel) and pooled data of at least three independent donors (lower panel) are shown. One-way ANOVA with Dunnett's multiple comparisons test. **D** Gene set variation analysis (GSVA) of MACE-seq results. *MLL*r cells and wt cells of at least two donors were analyzed. Colors indicate the *MLL* breakpoint, the fusion partner, and the donor. **E, F** Analysis of MACE-seq results. Significantly differentially expressed genes between cells with breakpoint in *MLL* intron 11 and *MLL* intron 9. Genes with p.adjust ≤ 0.05 were considered significant. Genes previously reported in the literature to be associated with leukemia and/or cancer are shown in the heatmap. **G, H** Analysis of MACE-seq results. Significantly differentially expressed genes between MLL::AF4 and MLL::AF9. Genes with p.adjust ≤ 0.05 were considered significant. Genes previously reported in the literature to be associated with leukemia and/or cancer are shown in the heatmap. **I** Mean IC_50_ values for cytarabine response in *MLL*r cells of three independent donors. One-way ANOVA with Dunnett's multiple comparisons test was performed to compare IC_50_ values to MLL(9)::AF9.

To characterize the CRISPR/Cas9-generated cells further, cytospins, followed by Pappenheim staining were performed. Compared to wt cells, *MLL*r cells exhibited a more immature morphology with a higher proportion of myeloblasts (Fig. 2A, Supplementary Figure S4).

Flow cytometry analysis revealed a myeloid immunophenotype in all *MLL*r cells, characterized by absent CD19 and high CD33 expression (Fig. 2B). All *MLL*r cells showed significantly elevated expression of the AML-related markers CD32, CD9, and CD123 [18,19]. The myeloid differentiation marker CD14 was significantly reduced in all *MLL*r cells compared to wt cells. CD117 (cKit), a marker for early leukemic and progenitor cells, was strongly elevated in MLL::AF9 cells but slightly decreased in MLL::AF4 cells (Fig. 2C).

MACE RNA-sequencing (-seq) was performed on all four *MLL*r cell types and wt cells of the same donors. Gene set variation analysis (GSVA) confirmed elevated MLL-related pathway activity in *MLL*r cells (Fig. 2D). The transcriptomic changes between *MLL::AF4* or *MLL::AF9* fusion and differences between the breakpoints were further analyzed (Supplementary Table S6). Comparison between the *MLL* breakpoints (*MLL* intron 9 or 11, MLL(9), MLL(11)) revealed minimal changes, with only 12-18 significantly differentially expressed genes (Fig. 2E, F). In contrast, 59-65 genes were differentially expressed between MLL::AF4 and MLL::AF9 (Fig. 2G, H). These findings suggest that the *MLL* fusion partner has a bigger influence on transcriptomic differences. The genes shown in heatmaps have established roles in cancer/leukemia progression and stemness, discussed further in the discussion section.

### *MLL*r cells are sensitive to cytarabine treatment

Chemotherapy remains a fundamental component of the standard AML therapeutic approach. We evaluated cytarabine responsiveness in all *MLL*r cells following 72-hour treatment with 0 nM to 1 µM cytarabine. Annexin/PI viability assays showed dose-dependent decreases in viable cells (PI⁻, Annexin V⁻) (Supplementary Figure S5). Based on calculations of dose-response curves, MLL(9)::AF9 cells had the highest IC_50_ values (Fig. 2I).

### After prolonged *in vitro* culture only MLL(9)::AF9 cells engraft in NSG mice

**Fig. 3:**
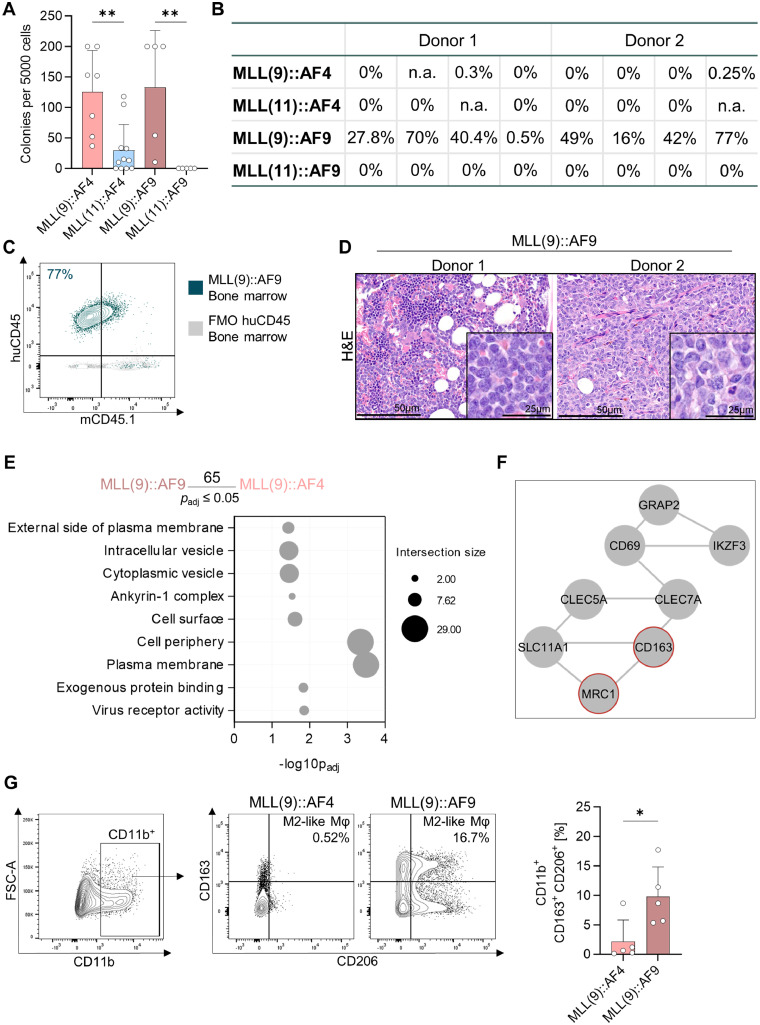
**A population of AML-supporting macrophages might promote engraftment of MLL(9)::AF9 cells. A** Colony counts of colony-forming unit assays with *MLL-*rearranged *(MLL*r) cells of three independent donors. One-way ANOVA with Šídák's multiple comparisons test. **B** Percentage of human cells (humanCD45^+^,murineCD45.1^-^, huCD45^+^, mCD45.1^-^) in the bone marrow (BM) of NSG mice. *MLL*r cells of two independent donors were transplanted. Percentages indicate engraftment levels in individual mice. n.a. no analysis possible. **C** Representative contour plot of flow cytometric assessment of huCD45^+^, mCD45.1^-^ cells in the BM of NSG mice. FMO, Fluorescence minus one control. **D** Hematoxylin and eosin (H&E) staining of BM sections (femur) of NSG mice. Images of the BM were taken at 40 × magnification, and at 60 × magnification (box in the corner). Representative images of one animal per donor are shown. Left: mouse ID #20, right: mouse ID #49. **E** Analysis of MACE-seq results revealed 65 significantly differentially expressed genes between MLL(9)::AF9 and MLL(9)::AF4 cells (p.adjust ≤ 0.05). These genes were used for pathway analysis. GO pathways are shown as a bubble plot indicating the intersection size and the -log10p_adj_. **F** Network topology-based analysis of genes with p_._adjust < 0.2 in WebGestalt. *MRC1* (CD206) and *CD163* are highlighted in red. **G** Flow cytometric analysis of MLL(9)::AF9 and MLL(9)::AF4 cells from myeloid *in vitro* culture to identify M2-like macrophages (Mφ) (CD11b^+^ CD163^+^ CD206^+^). Shown are representative plots and pooled data of five independent donors. Mann-Whitney test.

In colony-forming unit (CFU) assays, MLL(9) cells had a significantly higher colony count than MLL(11) cells (Fig. 3A). Notably, *HOXA9* expression patterns mirrored this clonogenic potential, with elevated expression levels in intron 9 versus intron 11 cells (Supplementary Figure S6).

We next transplanted *in vitro*-cultured *MLL*r cells of two independent huCB donors into NOD *scid* gamma (NSG) mice. Only MLL(9)::AF9 cells successfully engrafted in bone marrow (BM), as determined by flow cytometry detecting human CD45^+^ (huCD45^+^) and murine CD45.1^-^ (mCD45.1^-^) populations (Fig. 3B, C, Supplementary Figure S7A). Hematoxylin and eosin (H&E) staining of the BM confirmed leukemic infiltration, consistent with flow cytometric findings (Fig. 5D, Supplementary Figure S7B). Despite comparable *in vitro* colony-forming abilities between MLL(9)::AF4 and MLL(9)::AF9 (Fig. 5A), only MLL(9)::AF9 cells engrafted *in vivo*. We therefore reanalyzed MACE-seq data, comparing MLL(9)::AF9 and MLL(9)::AF4 cells (Supplementary Table S6). Pathway and network topology analyses of the 65 significantly differentially expressed genes in MLL(9)::AF9 samples revealed enrichment of cell surface-related pathways and high network connectivity of *CD163* and *MRC1* (encoding CD206) (Fig. 3E, F, Supplementary Figure S8A). These markers are expressed on tumor-supportive M2-like macrophages (M2-like Mφ) [20] and linked to enhanced engraftment, malignant transformation, and poor survival [21]. Flow cytometry confirmed significant enrichment of CD11b^+^ CD163^+^ CD206^+^ M2-like Mφ in MLL(9)::AF9 *in vitro* culture (Fig. 3G). In addition, *CD52*, previously associated with engraftment and tumor growth of AML [22], was higher expressed in MLL(9)::AF9 cells (Supplementary Figure S8A). Flow cytometry confirmed elevated CD52 surface expression *in vitro*, which potentially also contributed to superior MLL(9)::AF9 engraftment capacity (Supplementary Figure S8B).

### MLL(9)::AF9 cells retain lineage switch potential in NSG mice after 94 days of myeloid *in vitro* culture

**Fig. 4:**
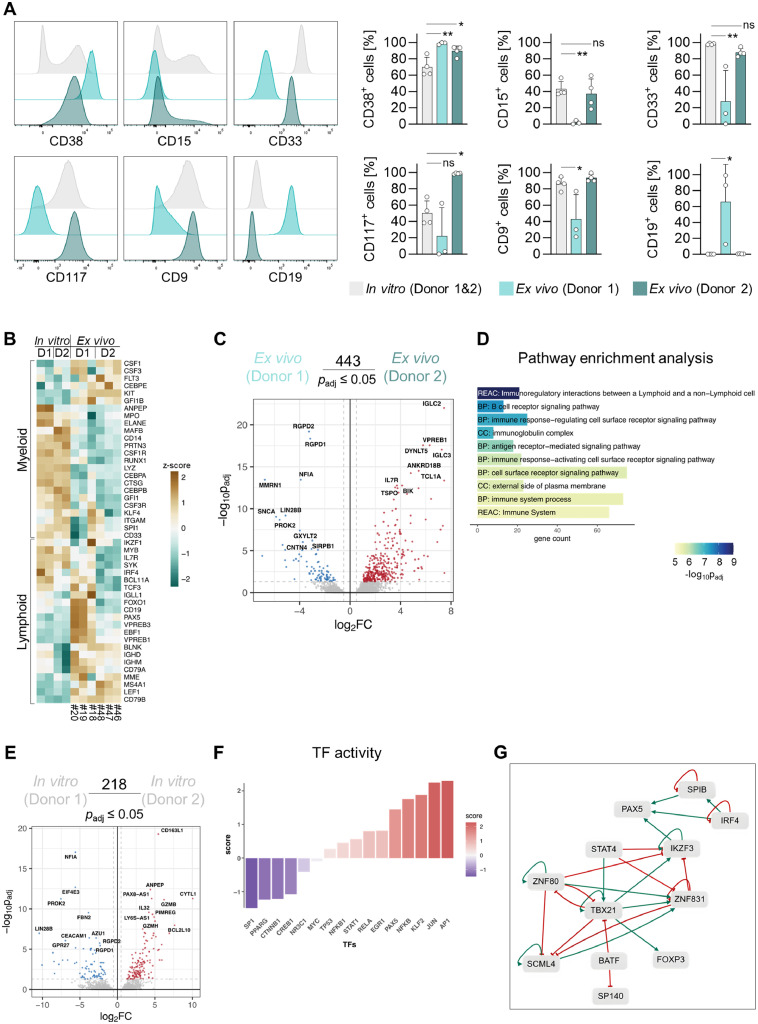
**MLL(9)::AF9 cells retain lineage switch potential in NSG mice after prolonged myeloid *in vitro* culture. A** Representative histograms (left panel) and quantification (right panel) of surface marker expression in humanCD45^+^, murineCD45.1^-^ (huCD45^+^, mCD45.1^-^) cells isolated of the bone marrow (BM) of NSG mice. The *in vitro* group includes pooled data of Donor 1 and Donor 2 of repeated measures. *Ex vivo* data of Donor 1 and Donor 2 represent pooled measurements of at least three individual recipient mice per donor. One-way ANOVA with Dunnett's multiple comparisons test. **B** RNA-seq was performed on huCD45^+^, mCD45.1^-^ cells isolated of the BM of NSG mice. Heatmap with z-scores displaying gene clusters associated with myeloid and lymphoid lineages. The *in vitro* group includes pooled data of Donor 1 and Donor 2 (D1, D2) of two different frozen batches. *Ex vivo* data of Donor 1 and Donor 2 represent pooled measurements of three individual recipient mice per Donor (mouse IDs at the bottom). **C** Differentially expressed genes between *ex vivo* samples of Donor 1 and Donor 2. Genes with a log2FC ≥ 0.5 and p.adjust ≤ 0.05 were considered significant. **D** Pathway analysis of genes significantly differentially expressed genes in Donor 1 (*in vivo*) relative to Donor 2 (*in vivo*). **E** 218 genes were significantly differentially expressed between *in vitro* samples of Donor 1 compared to Donor 2. Genes with a log2FC ≥ 0.5 and p.adjust ≤ 0.05 were considered significant. **F** Activity prediction of different transcription factors (TFs) in Donor 1 relative to Donor 2 (*in vitro*). **G** TF network with activation links (green) and inhibition links (red). The 218 differentially expressed genes in *in vitro* samples of Donor 1 compared to Donor 2. (visualized in E) were used as input.

To explore how a physiological microenvironment influenced the MLL(9)::AF9 cells, we analyzed the engrafted cells in the BM of NSG mice and *in vitro* cultured cells of the same donor. Flow cytometric immunophenotyping revealed that Donor 1 MLL(9)::AF9 cells exhibited significantly reduced CD15, CD33, and CD9 expression compared to *in vitro*-cultured cells, accompanied by marked CD19 upregulation, indicating lineage switch from myeloid to B-lymphoid identity *in vivo*. Conversely, Donor 2 cells retained myeloid marker expression and remained CD19^-^ (Fig. 4A).

To analyze the transcriptome of *ex vivo* cells by RNA-seq, we performed fluorescence-activated cell sorting and isolated human MLL(9)::AF9 cells (huCD45^+^, mCD45.1^-^) from the BM of NSG mice (Supplementary Table S7, Supplementary Figure S9A). Obtained transcriptomic signatures aligned with immunophenotypic profiles: Lymphoid genes and transcription factors (TFs) were highly expressed in Donor 1 *ex vivo* cells (Fig. 4B), with *CD19, KIT* (encoding CD117), and *CD33* expression matching flow cytometric data (Supplementary Figure S9B). Between Donor 1 and Donor 2 *ex vivo* samples, 443 genes were differentially expressed, and pathway enrichment analysis revealed B-cell pathway activation in Donor 1 (Fig. 4C, D).

To understand why only Donor 1 underwent lineage switch, we compared the *in vitro* samples and found 218 genes to be significantly differentially expressed (Fig. 4E). Lymphoid TFs, like *PAX5* and *AP1*, showed higher expression and predicted activity in Donor 1, while predicted activity of myeloid TFs, like SP1 and CREB1 was reduced. (Figure E, F, Supplementary Figure S9C). Transcription factor enrichment analysis confirmed that Donor 1 had established a stable lymphoid TF network *in vitro*, with IKZF3 (AIOLOS) and PAX5 being central players (Fig. 4G). Involvement of TBX21, BATF, and STAT4 suggested a plastic transcriptional state, where the B-lymphoid program is not fully stabilized.

### Individual mice transplanted with Donor 1 represent distinct stages of B-cell differentiation

**Fig. 5:**
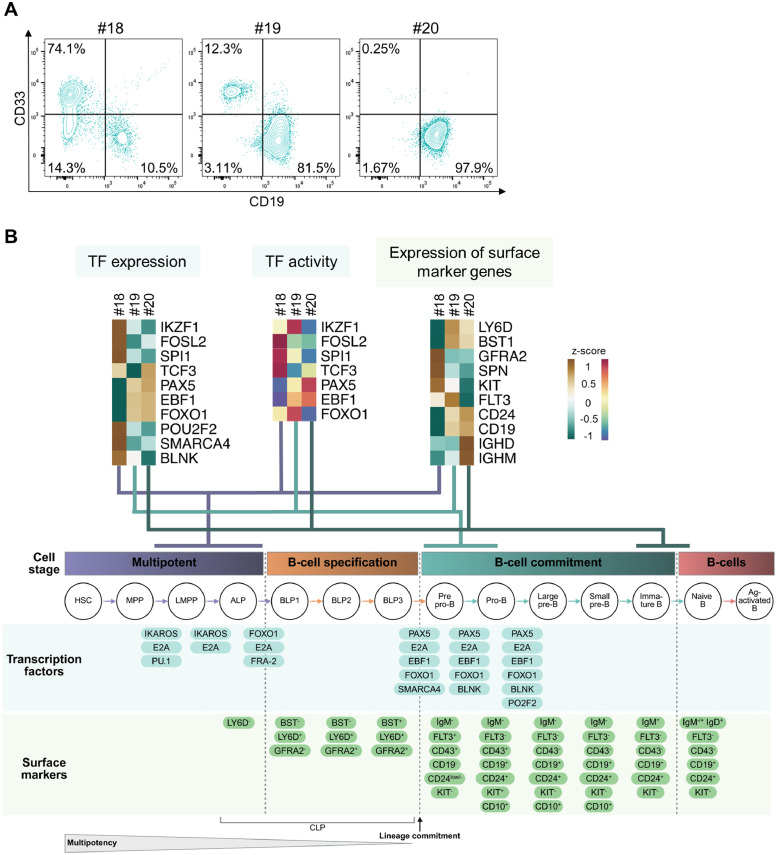
**Individual mice transplanted with Donor 1 represent distinct stages of B-cell differentiation. A** Contour plots of flow cytometric analysis showing CD33, CD19 (co-)expression in individual NSG mice labeled #18, #19, and #20. All mice were transplanted with MLL(9)::AF9 cells of Donor 1. **B** Gene expression of different transcription factors (TFs) and surface markers and predicted TF activity in RNA-seq data of mouse #18, #19 and #20. HSC, hematopoietic stem cells; MPP, multipotent progenitors; LMPP, lymphoid primed MPPs; ALP, all-lymphoid progenitors; BLP, B-cell-biased lymphoid progenitors; CLP, common lymphoid progenitors. Figure modified from Fischer et al. [23]. Figure created with Biorender.com.

CD19 expression on MLL(9)::AF9 cells of Donor 1 varied substantially across recipient mice (Fig. 4A). Only ∼12% of human cells of mouse #18 expressed CD19, whereas human cells of mice #19 and #20 were 86-99% CD19^+^. Notably, cells displayed distinct CD19^+^ or CD33^+^ populations rather than a mixed lineage phenotype (CD33^+^ CD19^+^) (Fig. 5A). RNA-seq data revealed differential expression of TFs and lineage-defining surface markers, suggesting that mice #18, #19, and #20 represent progressive stages of B-cell lineage commitment. To contextualize these observations, we mapped our samples to a “timeline” of B-cell differentiation based on Fischer et al. [23] (Fig. 5B). Mouse #18 displayed features of a multipotent cell on the way to B-cell specification, as FRA2 (*FOSL2*), PU.1 (*SP1*) and IKAROS (*IKZF1*) expression and predicted activity were high. Mouse #19 showed a transcriptional profile consistent with a pre-pro-B to pro-B-cell-stage, marked by high FOXO1 activity and increased gene expression of *FLT3, CD24*, and *CD19*. Finally, mouse #20 expressed *IGHD* (IgD) and *IGHM* (IgM), representing a fully committed B-cell identity.

### *In vivo*, KRAS signaling is activated in MLL(9)::AF9 cells independently of *KRAS* mutations

**Fig. 6:**
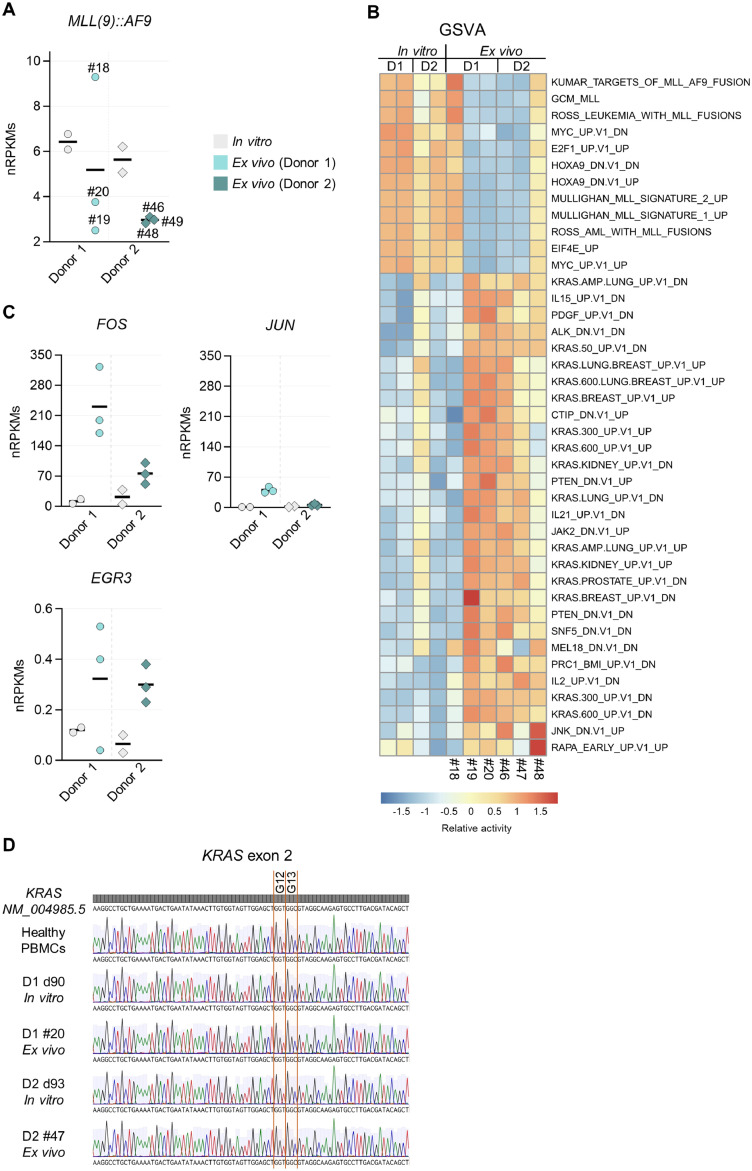
***In vivo* MLL(9)::AF9 cells activate KRAS signaling independently of KRAS mutations. A** MLL(9)::AF9 cells cultured *in vitro* and sorted huCD45^+^ mCD45.1^-^*MLL*r cells of the bone marrow (BM) of NSG mice (*ex vivo*) were analyzed by RNA-seq. The *in vitro* group includes pooled data of Donor 1 and Donor 2 of two different frozen batches. *Ex vivo* data of Donor 1 and Donor 2 represent pooled measurements of at least three individual recipient mice per Donor. Normalized Reads Per Kilobase per Million mapped reads (nRPKMs) of the *MLL(9)::AF9* transcript are shown. Individual recipient mice are labeled with the mouse IDs. **B** Gene set variation analysis (GSVA) using RNA-seq data of *in vitro* cultured MLL(9)::AF9 cells and sorted *MLL*-rearranged cells of the BM of NSG mice (*ex vivo*). Mouse IDs at the bottom. D, Donor. **C** Normalized RPKMs of *FOS, JUN* and *EGR3*. **D** Detection of potential *KRAS* mutations in exon 2 by genomic PCR and Sanger sequencing. *KRAS* mutation hotspots codon 12 and 13 are highlighted in orange (G, glycine). From upper to lower row: *KRAS* reference sequence (RefSeq: NM_004985.5); PBMCs (healthy donor); *in vitro* cultured cells Donor 1, 90 days after translocation induction; *ex vivo* cells of mouse #20, (transplanted with Donor 1); *in vitro* cultured cells Donor 2, 93 days after translocation induction; *ex vivo* cells of mouse #47 (transplanted with Donor 2). D, donor; d, day.

As MLL oncogene expression levels can vary and influence disease progression [24], we quantified the *MLL(9)::AF9* expression in our RNA-seq data of *in vitro* and *ex vivo* MLL(9)::AF9 samples (Fig. 6A). We found that *MLL(9)::AF9* expression was overall low *in vivo*, except for mouse #18 (Donor 1), which showed the highest expression levels*. In vitro, MLL(9)::AF9* expression was increased (up to 2.7-fold higher), but still below the level observed in mouse #18. To determine whether reduced MLL-fusion program activity in Donor 2 *ex vivo* resulted from myeloid differentiation, we analyzed CD14 expression by flow cytometry. CD14 was not expressed on the *ex vivo* cells (Supplementary Figure S10A), indicating that low *MLL(9)::AF9* expression was unlikely due to differentiation.

By GSVA, we assessed whether *MLL(9)::AF9* expression patterns correlated with downstream MLL-related pathway activity. All *in vitro* samples and mouse #18 and #48 showed high activity of MLL-driven pathways, including MYC and HOXA9 targets (Fig. 6B). Remaining *ex vivo* samples showed reduced activity of these programs, but together with mouse #48, they exhibited activation of multiple KRAS-associated gene sets. Supporting this, the genes encoding for the TFs FOS and JUN, well-established downstream effectors of KRAS-ERK pathway activation, were upregulated in the *ex vivo* samples [25,26] (Fig. 6C). Furthermore, expression of *EGR3* (encoding a C2H2 zinc finger transcription factor) was elevated *in vivo*.

Since KRAS mutations are common in *MLL*r leukemias, we performed genomic PCR and Sanger sequencing to detect potential mutations [27]. We screened exons 2 and 3, known mutation hotspots [28], but detected no mutations in these regions (Fig. 6D, Supplementary Figure S10B), suggesting that enhanced KRAS-signaling activity arises from microenvironmental stimuli or altered upstream RAS regulation.

## Discussion

We successfully generated *MLL*r cells harboring *MLL::AF4* or *MLL::AF9* fusions with breakpoint in intron 9 and extended our previous intron 11 model, enabling for the first time the direct comparison of different *MLL* breakpoints and fusion partners within the same donor. Comparative analysis of the immunophenotype revealed that all *MLL*r cells upregulated AML, stemness, and LSC markers like CD123, CD32, and CD9, and downregulated the differentiation marker CD14^18, 19^. MLL::AF9 cells exhibited higher CD117 expression compared to wt cells, potentially reflecting their myeloid progenitor identity. It could be suggested that this marker for myeloid progenitor cells cannot be upregulated by MLL::AF4 cells due to their tendency to differentiate towards the B lymphoid lineage, where CD117 is not expressed [29].

MACE-seq analysis revealed that both the *MLL* breakpoint location and especially the fusion partner significantly shape the leukemic transcriptional landscape. Key hematopoietic regulators and lineage-defining factors, including *PBX3, ZNF521, TAL1*, and *NFIA*, showed differential expression patterns [[30], [31], [32], [33]]. Epigenetic regulators (*EP300, SMYD3*), stemness- and/or prognosis-associated genes (*SOCS2, THBS1, IGF2BP1*), and drug resistance markers (*ABCC3*) varied by translocation type, potentially explaining functional differences such as cytarabine sensitivity [[34], [35], [36], [37], [38], [39]]. The non-coding RNAs *LINC00536* and *LINC00273*, known for their role in tumor progression and metastasis in solid tumors, were expressed differently depending on the breakpoint [40,41]. Although these results need functional confirmation, the findings highlight the influence of the breakpoint location and especially the *MLL* fusion partner. As these factors may influence disease aggressiveness and response to therapy, continued investigation may identify novel therapeutic targets.

In CFU assays, MLL(9) cells had a higher self-renewal capacity than cells with breakpoint in *MLL* intron 11. This correlates with elevated *HOXA9* expression, a known factor of *MLL*r leukemogenesis and clonogenic potential [42]. The lack of MLL(11)::AF9 engraftment (contrasting our TALEN model [43]) likely reflects its reduced leukemogenic potential under the current experimental conditions. Our 52-week observation period represented the lower limit for detecting disease, given the 48–60 week latency reported previously [43]. Moreover, the shift from physiologically tailored human plasma to standardized serum-based culture likely compromised the maintenance of leukemia-initiating cells. These factors underscore the superior oncogenic drive of MLL(9)::AF9 cells, which remains robust despite more restrictive conditions. Beyond these intrinsic factors, we identified a subpopulation of M2-like Mφ among the MLL(9)::AF9 cells. AML-derived Mφ enhance homing and leukemia development *in vivo*, and confer chemotherapy resistance [21,44]. This aligns with our observation that MLL(9)::AF9 cells had the highest IC_50_ in response to cytarabine. We also found CD52 to be elevated on MLL(9)::AF9 cells. Reduction of AML tumor growth and block of engraftment in mice treated with the anti-CD52 antibody Alemtuzumab have already been shown [22]. Whether CD52 could be a promising target in MLL(9)::AF9 AML will be investigated in the future.

In contrast, MLL(9)::AF4 cells may be disadvantaged in their ability to home and engraft due to impaired formation of the reciprocal *AF4::MLL* fusion. The sgRNA targeting *AF4* exon 3, which was designed according to a patient sequence [45], may have disrupted splicing or induced non-functional isoforms, resulting in absent or insufficient *AF4::MLL* mRNA despite genomic detection. Although AF4::MLL appears dispensable in established t(4;11) leukemia, early expression may be critical for overwriting transcriptional elongation control and establishing active chromatin [11,46]. Without cooperative MLL::AF4 and AF4::MLL effects, leukemic transformation is attenuated, potentially explaining engraftment failure in our setting. Furthermore, MLL::AF9′s predominant AML association may preserve stem cell-like properties and engraftment capacity in myeloid culture (indicated by high CD117), whereas MLL::AF4′s ALL association may render cells prone to differentiation or dysregulation affecting niche interactions under myeloid culture conditions. Furthermore, *in vitro* immortalization does not necessarily predict engraftment *in vivo* [47]. This might be particularly evident for MLL::AF4, as the developmental ontogeny profoundly influences the leukemic potential of the cells. Secker et al. [48] demonstrated that HSPCs from neonatal sources are susceptible to malignant transformation by MLL::AF4, while adult BM cells remain resistant. Rice et al. [49] further showed that fetal liver provides an even more permissive landscape by utilizing fetal-specific programs, such as the LIN28B/HMGA2 axis, which decline as cells transition from a fetal to a neonatal state. We postulate that our prolonged myeloid culture (94 days) further exhausted these declining fetal signals, abrogating the oncogenic potential *in vivo*. Using our approach, Benz et al. demonstrated in unpublished data that *MLL*r cells with t(4;11) and t(6;11) chromosomal translocations engraft rapidly when transplanted immediately after translocation induction [50]. This suggests that immediate transfer to the *in vivo* niche prevents culture-induced exhaustion and the exhaustion of MLL::AF4 cooperative fetal/neonatal programs. Earlier transplantation will therefore result in valuable mouse models, successfully mimicking the disease and allowing for a comparison of different *MLL* fusions *in vivo*.

Remarkably, MLL(9)::AF9 cells retained robust engraftment capacity despite prolonged *in vitro* culture. Donor 1 cells cultured under myeloid conditions for 94 days underwent lineage switch from myeloid to B-lymphoid upon xenotransplantation. While Barabé et al. reported that MLL::AF9 cells transplanted after 30 days could result in AML or B-ALL, later transplantation led to AML or no engraftment [51]. In our study, the cells retained their lineage switch capacity even after prolonged myeloid *in vitro* culture (>90 days). Similar to the observations of Mulloy et al., who proposed that the cytokine cocktail might alter the lifespan of suspension cells *in vitro*, we suspect that our culture conditions might have kept the cells in a more pluripotent state, compared to the experimental setup of Barabé et al. [47]. Interestingly, the lineage switch capacity was donor-specific. This donor-dependent variability represents a key strength of our model, capturing clinically relevant patient-to-patient heterogeneity.

*In vivo*, the BM microenvironment provides a complex network of signals. Our cells displayed reduced *MLL(9)::AF9* transcript levels and decreased activation of MLL-associated signaling pathways *in vivo*. At the same time, KRAS-signaling was activated, seemingly providing a selective advantage that rendered high *MLL::AF9* expression dispensable, and might have led to its downregulation. Assuming no other genetic alterations are contributing to these changes, these observations highlight the influence of the physiological niche on oncogenic signaling. It will be interesting to see if these effects also occur when the cells are transplanted directly after translocation induction, as long-term *in vitro* culture might have selected a KRAS-dependent subclone that emerged *in vivo*.

In conclusion, our study provides a strong foundation for investigating how different *MLL* fusion partners and the *MLL* breakpoint influence leukemia biology. We established a fast and efficient system in which both reciprocal fusion proteins are generated and expressed under physiological conditions. Importantly, this strategy can be extended to HSPCs of different origins, such as BM or fetal liver, allowing modeling disease heterogeneity further [16,49]. This powerful, flexible platform advances mechanistic understanding of *MLL*r leukemia *in vitro* and *in vivo* and supports targeted therapy evaluation in contexts closely mirroring patient disease biology.

## Note on nomenclature

The historical terms *MLL, AF9*, and *AF4* are used for *KMT2A, MLLT3*, and *AFF1*, respectively, primarily for brevity and readability.

## Data availability

RNA-seq data files have been uploaded to the Gene Expression Omnibus (GEO) database and are available under the accession numbers GSE311674 (MACE-seq) and GSE311933 (RNA-seq). Additional data supporting the findings of this study are available from the corresponding author upon reasonable request and in the supplementary material of this article.

## CRediT authorship contribution statement

**Pia Radszuweit:** Writing – review & editing, Writing – original draft, Visualization, Validation, Project administration, Investigation, Formal analysis, Data curation, Conceptualization. **Rahel Fitzel:** Writing – review & editing, Project administration, Methodology, Investigation, Formal analysis, Conceptualization. **Sarah Bruestl:** Writing – review & editing, Investigation, Formal analysis. **Thomas Hentrich:** Writing – review & editing, Visualization, Software, Formal analysis, Data curation. **Fulya Korkmaz:** Writing – review & editing, Investigation. **Barbara Mankel:** Writing – review & editing, Investigation, Formal analysis. **Irene González-Menéndez:** Writing – review & editing, Visualization, Validation, Investigation, Formal analysis. **Saskia Rudat:** Writing – review & editing, Investigation. **Rolf Marschalek:** Writing – review & editing, Conceptualization. **Estelle Erkner:** Writing – review & editing, Investigation. **Hildegard Keppeler:** Writing – review & editing, Investigation. **Rebekka Schairer:** Writing – review & editing, Investigation. **Luise Luib:** Writing – review & editing, Investigation. **Markus Mezger:** Writing – review & editing, Resources, Investigation. **Leticia Quintanilla-Martinez:** Writing – review & editing, Validation, Resources, Formal analysis. **Julia Schulze-Hentrich:** Writing – review & editing, Resources. **Claudia Lengerke:** Writing – review & editing, Resources, Funding acquisition. **Dominik Schneidawind:** Writing – review & editing, Supervision, Resources, Funding acquisition. **Corina Schneidawind:** Writing – review & editing, Validation, Supervision, Resources, Project administration, Funding acquisition, Data curation, Conceptualization.

## Declaration of competing interest

The authors declare the following financial interests/personal relationships which may be considered as potential competing interests:

Given her role as Associate Editor for Blood Advances, Claudia Lengerke had no involvement in the peer-review of this article and has no access to information regarding its peer-review. Full responsibility for the editorial process for this article was delegated to another journal editor. The other authors have no known competing financial interests or personal relationships that could have appeared to influence the work reported in this paper.

## Appendix Supplementary materials

Image, application 1

Image, application 2

Image, application 3
