# Supplementary material for: Characterizing the impact of MLL fusion variants and fusion partners on leukemia plasticity using a human CRISPR-engineered MLL-rearranged leukemia model
Source: Neoplasia. 2026 Apr 18;77:101308. doi: 10.1016/j.neo.2026.101308 (PMC13098342; doi:10.1016/j.neo.2026.101308)
Supplement: Supplementary file 1 [file mmc1.pdf]

Supplementary material for

Characterizing the Impact of *MLL* Fusion Variants and Fusion Partners on Leukemia Plasticity Using a Human CRISPR-Engineered *MLL*-Rearranged Leukemia Model

Pia Radszuweit[1, 2], Rahel Fitzel[2], Sarah Bruestl[2], Thomas Hentrich[3], Fulya Korkmaz[2], Barbara Mankel[4], Irene González-Menéndez[4, 5], Saskia Rudat[2], Rolf Marschalek[6], Estelle Erkner[2], Hildegard Keppeler[2], Rebekka Schairer[2], Luise Luib[7], Markus Mezger[7], Leticia Quintanilla-Martinez[4, 5, 8], Julia Schulze-Hentrich[3], Claudia Lengerke[2], Dominik Schneidawind[1, 2], Corina Schneidawind[1, 2]

[1]*Department of Medical Oncology and Hematology, University Hospital Zurich, Zurich, Switzerland*

[2]*Department of Internal Medicine II, University Hospital Tübingen, Tübingen, Germany*

[3]*Department of Genetics/Epigenetics, Saarland University, Saarbrücken, Germany*

[4]*Institute of Pathology and Neuropathology and Comprehensive Cancer Center, University Hospital Tübingen, Tübingen, Germany*

[5]*Core Facility Histology, Faculty of Medicine, University of Tübingen, Tübingen, Germany*

[6]*Institute of Pharmaceutical Biology, Goethe-University Frankfurt, Frankfurt/Main, Germany*

[7]*Department of General Paediatrics, Haematology/Oncology, University Children's Hospital Tübingen, Tübingen, Germany*

[8]*Cluster of Excellence iFIT (EXC 2180) "Image-Guided and Functionally Instructed Tumor Therapies", Eberhard Karls University, Tübingen, Germany*

The PDF file includes:

Supplementary figure S1-S10

Supplementary methods

Supplementary tables S1-S5

Supplementary reference list

Supplementary figures

Supplementary figure S1

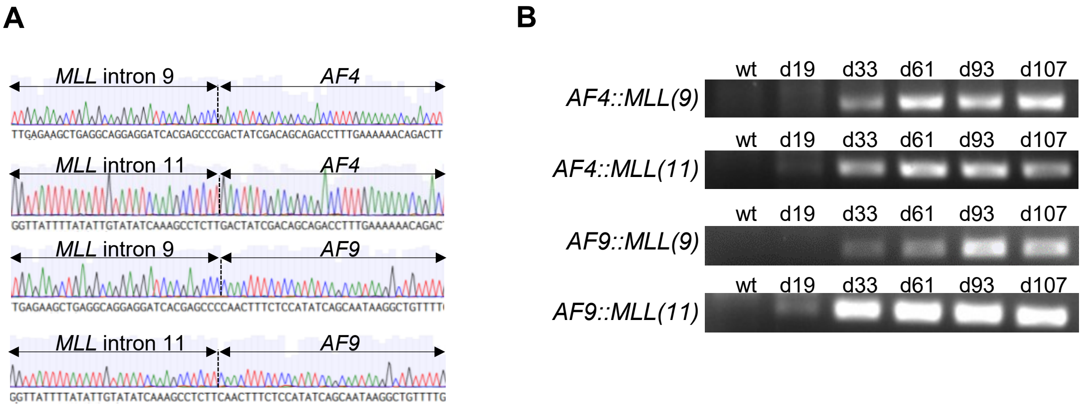

Supplementary Figure S1: A Sanger sequencing of the *MLL::AF4* or *MLL::AF9* genomic fusion sites. Results of one representative donor are shown. **B** Detection of the reciprocal fusion of *AF4* or *AF9* with *MLL* intron 9 or 11 in CRISPR/Cas9-modified cells by genomic PCR 19, 33, 61, 93, and 107 days (d) after translocation induction. Results of one representative donor are shown.

Supplementary figure S2

wt

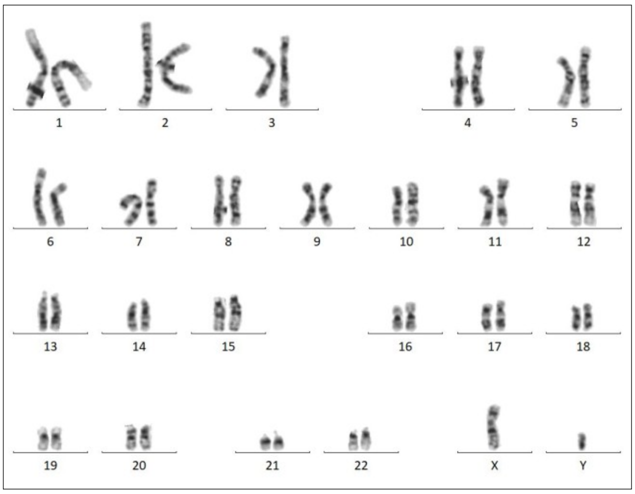

t(4;11)

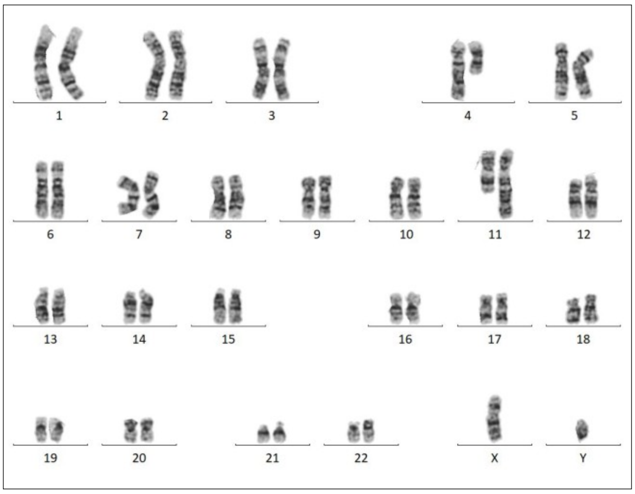

t(9;11)

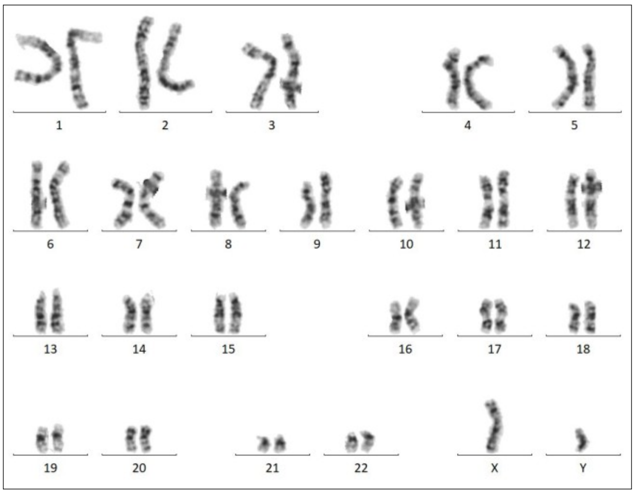

Supplementary Figure S2: Complete G-banded karyotypes of wt and *MLL*r cells of one representative donor. t(4;11) includes cells with a fusion of *MLL* and *AF4*. t(9;11) includes cells with a fusion of *MLL* and *AF9*.

Supplementary figure S3

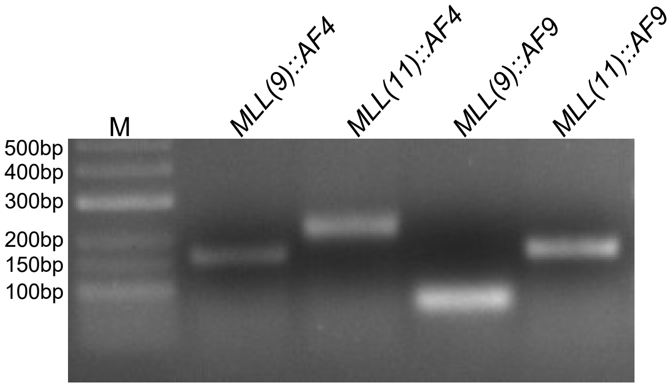

Supplementary Figure S3: Detection of the *MLL::AF4* and *MLL::AF9* fusion transcripts in CRISPR/Cas9-modified *MLL*r cells by RT-PCR. Gel images with the separated RT-PCR products of one representative donor are shown. M, marker. bp, base pairs.

Supplementary figure S4

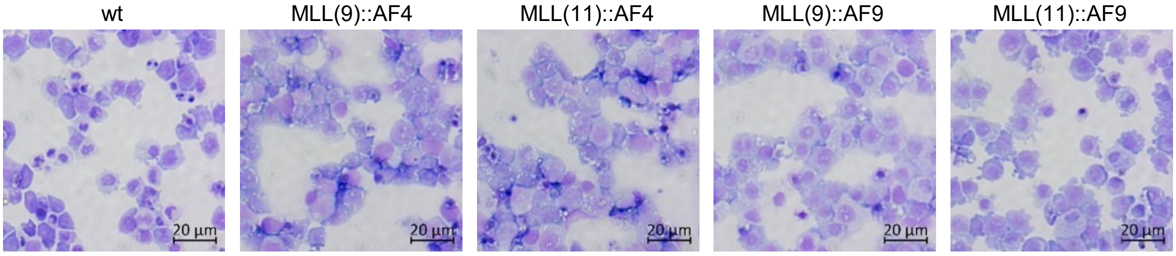

Supplementary Figure S4: Representative cell morphologies of *MLL*r and wildtype (wt) cells. Cells were stained by Pappenheim staining. Scale bar indicates 20 µm.

Supplementary figure S5

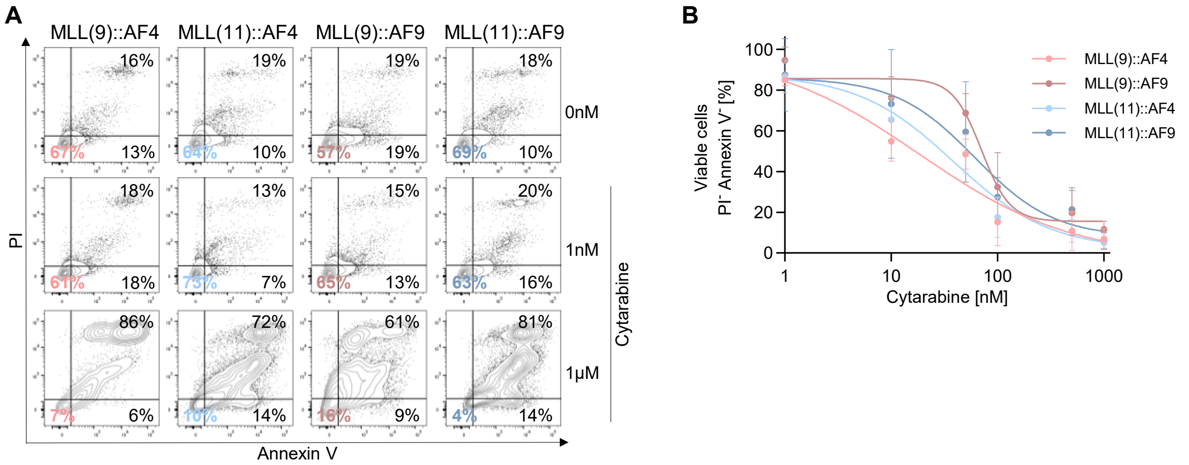

Supplementary Figure S5: A Representative contour plots of flow cytometric analysis of Annexin V/PI staining of *MLL*r cells after treatment with cytarabine for 72 hours. Percentage of viable cells (PI$^-$, Annexin V$^-$, in color) was used to determine the response to cytarabine. **B** Dose response curve of *MLL*r cells of three independent donors after treatment with 0 nM, 1 nM, 10 nM, 50 nM, 00 nM, 500 nM, and 1 µM cytarabine for 72 hours. Percentage of viable cells was determined by Annexin V/PI staining.

Supplementary figure S6

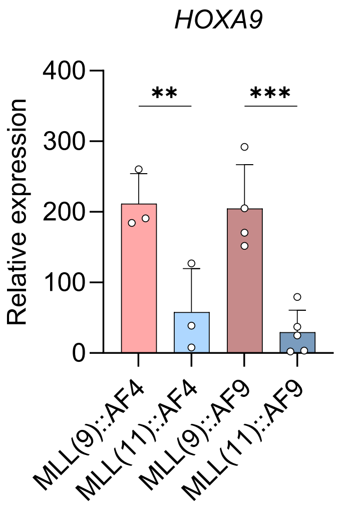

Supplementary Figure S6: Relative *HOXA9* expression in *MLL*-rearranged cells compared to wildtype cells measured by qRT-PCR. Pooled data of at least 3 independent donors presented as mean with SD. One-way ANOVA with Šídák's multiple comparisons test. **p ≤ 0.01, *** p ≤ 0.001.

Supplementary figure S7

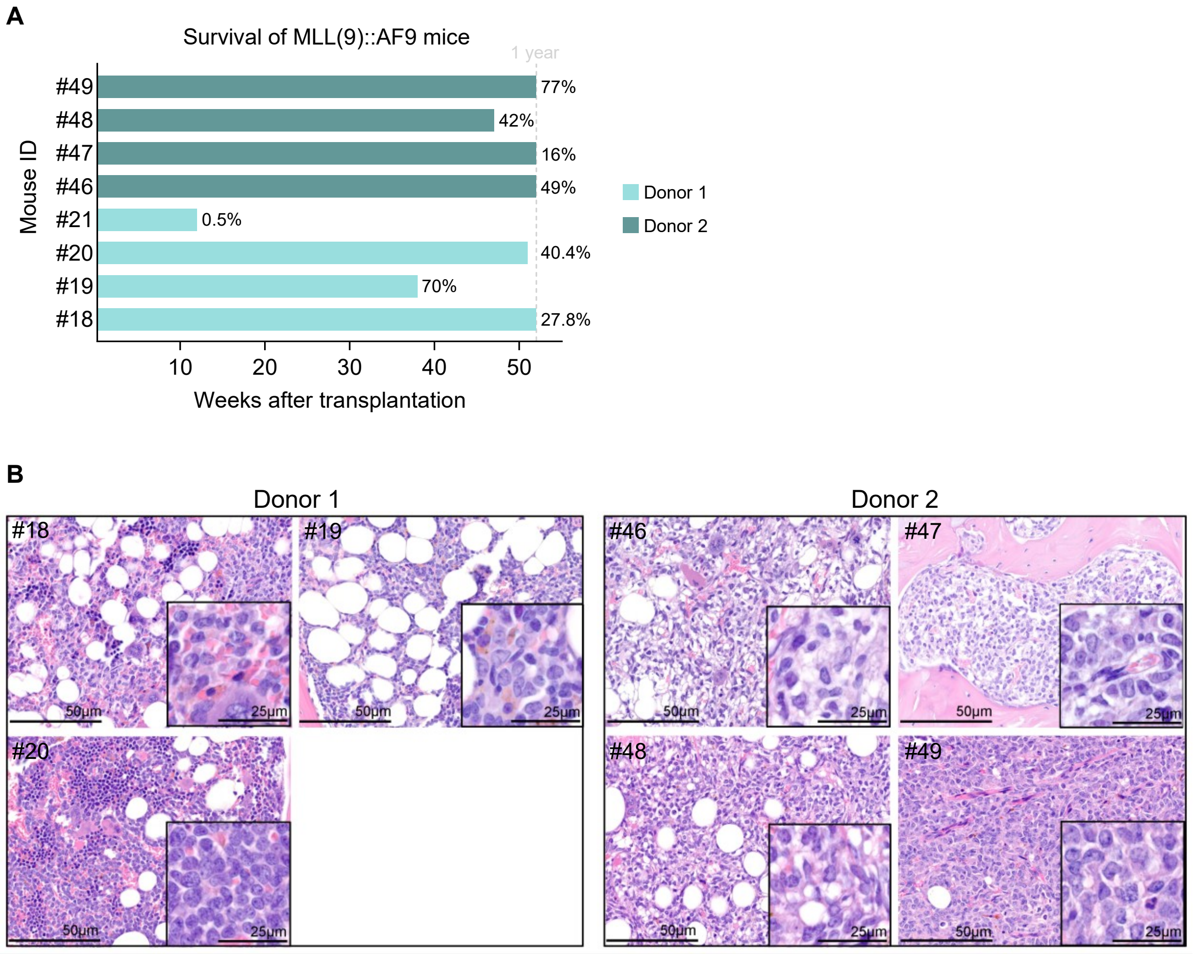

Supplementary Figure S7: A Survival of mice transplanted with MLL(9)::AF9 cells of Donor 1 and 2, and engraftment of human cells in the bone marrow (BM) of the respective NSG mouse. Length of the bars indicates the survived weeks. Dotted gray line indicates the maximal follow up time of 1 year. Mouse ID, mouse identity number. **B** Hematoxylin and eosin (H&E) staining of bone marrow (femur) sections from all NSG mice transplanted with MLL(9)::AF9 cells. Images of the bone marrow were taken at 40× magnification, and at 60× magnification (box in the corner).

Supplementary figure S8

A

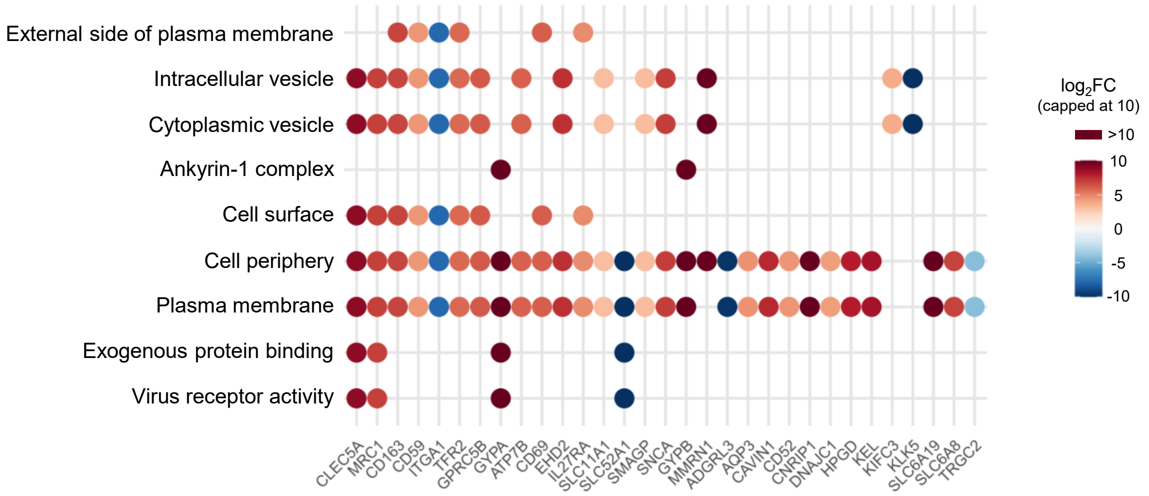

B

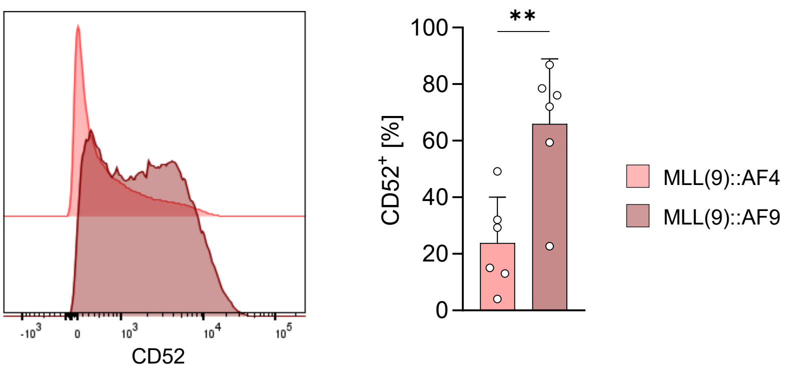

Supplementary Figure S8: A Analysis of MACE-seq results revealed 65 significantly differentially expressed genes between MLL(9)::AF9 and MLL(9)::AF4 cells (p.adjust ≤ 0.05). These genes were used for pathway analysis by g:profiler. Abundance of genes in the respective GO pathways are shown. Log_2 fold changes are indicated as a heatmap. Log_2FC values above 10 were capped to maintain the dynamic range of the majority of data. **B** Flow cytometric analysis of 100% pure MLL(9)::AF9 and MLL(9)::AF4 cells for CD52 surface expression *in vitro*. Shown are representative plots and pooled data of 6 independent donors presented as mean with SD. Unpaired t test. **p ≤ 0.01.

Supplementary figure S9

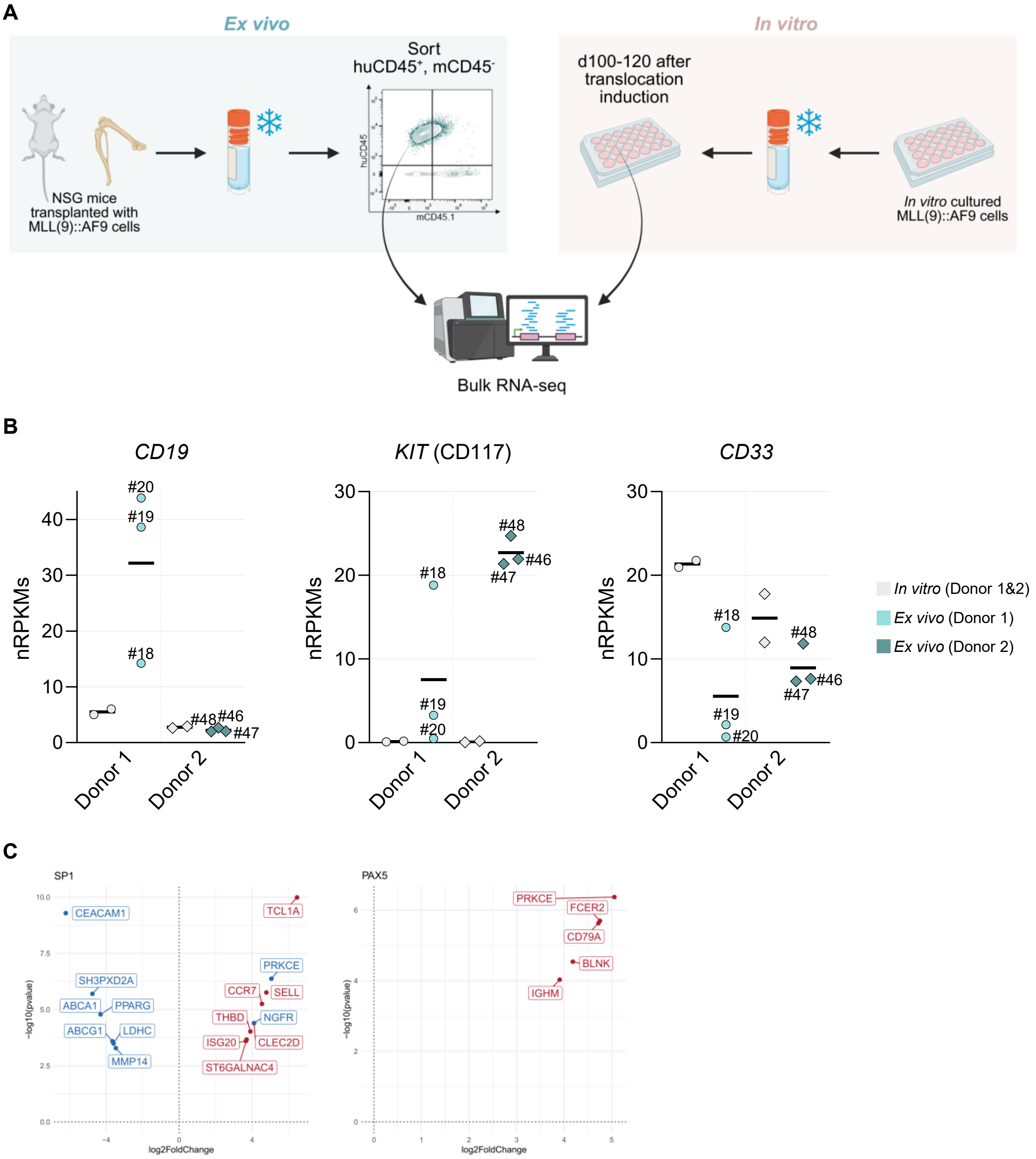

Supplementary Figure S9: A Schematic representation of experimental design of bulk RNA-seq to analyze *in vitro*-cultured MLL(9)::AF9 cells and *ex vivo* MLL(9)::AF9 cells of the same human donors, isolated from the bone marrow (BM) of NSG mice. d, day. Figure created with Biorender.com. **B** MLL(9)::AF9 cells from *in vitro* culture and sorted $huCD45^{+}$, $mCD45^{-}$ (hu, human; m, murine) MLLr cells from the BM of NSG mice (*ex vivo*) were analyzed by RNA-seq. Normalized RPKMs of genes encoding CD19, CD117 (*KIT*), and CD33 are shown. The *in vitro* group includes pooled data from Donor 1 and Donor 2 from two different frozen batches. *Ex vivo* data of Donor 1 and Donor 2 represent pooled measurements from at least three individual recipient mice per Donor. Black lines indicate the mean of each sample set. **C** Log_2FC of SP1 and PAX5 activating and deactivating genes in Donor 1 relative to Donor 2. Target genes expected to deactivate the respective transcription factors (TFs) are highlighted in blue, whereas genes expected to activate the respective TF are shown in red.

Supplementary figure S10

A

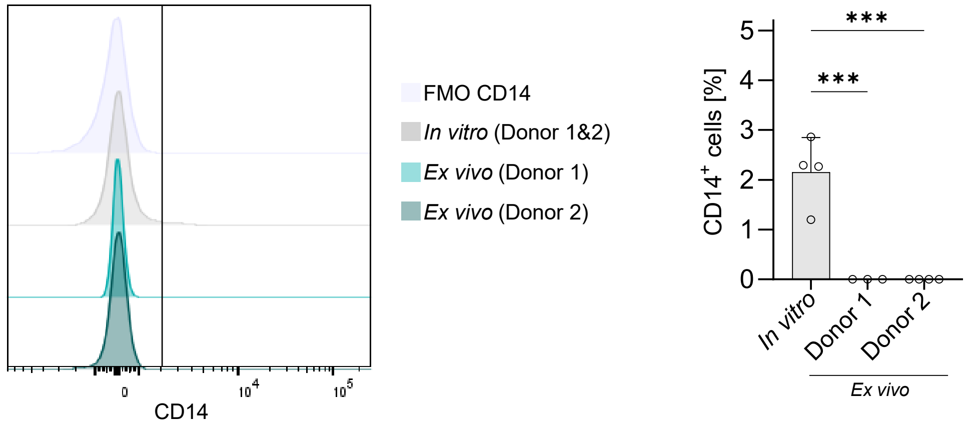

B

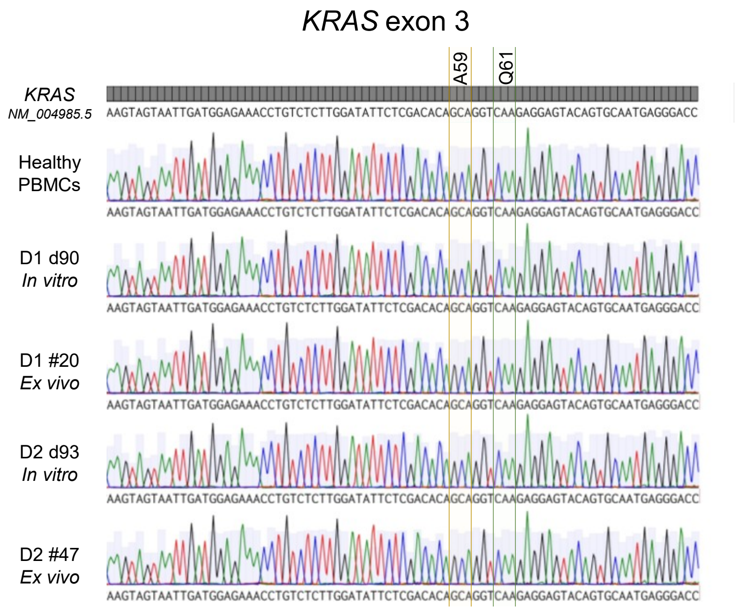

Supplementary Figure S10: A Representative histogram and quantification showing surface marker expression of CD14 in huCD45^{+}/mCD45^{-} (hu, human; m, murine) cells isolated from the bone marrow (BM) of NSG mice. Data are shown for Donor 1 and Donor 2 (MLL(9)::AF9) and compared to *in vitro*-cultured cells of the same donors. The CD14^{+} population was gated according to the FMO (fluorescence minus one control). The *in vitro* group includes pooled data of Donor 1 and Donor 2, each measured in technical duplicates. *Ex vivo* data of Donor 1 and Donor 2 represent pooled measurements of at least three individual recipient mice per donor. Data are shown as mean with SD. One-way ANOVA with Dunnett's multiple comparisons test. *** $p \leq 0.001$. **B** Detection of *KRAS* mutations in exon 3 by genomic PCR followed by Sanger sequencing. The *KRAS* mutation hotspots codon 59 and 61 are highlighted in yellow and green (A, alanine; Q, glutamine). From upper to lower row: *KRAS* reference sequence (RefSeq: NM_004985.5); PBMCs from a healthy donor; *in vitro*-cultured cells of Donor 1, 90 days after translocation induction; *ex vivo* cells of mouse #20, transplanted with cells of Donor 1; *in vitro*-cultured cells of Donor 2, 93 days after translocation induction; *ex vivo* cells of mouse #47, transplanted with cells of Donor 2. D, donor; d, day.

Supplementary methods

RT-PCR

RNA was isolated using the NucleoSpin RNA Kit (Macherey-Nagel, Cat# 740955.50) according to manufacturer's instructions. For reverse transcription of RNA to cDNA, the RevertAid H Minus First Strand cDNA Synthesis Kit (Thermo Fisher Scientific, Cat# K1631) was used according to manufacturer's instructions. *MLL* fusion transcripts were amplified using Maxima SYBR Green qPCR Master Mix (Thermo Fisher Scientific, Cat# K0222). PCR products were separated on a 2.5% agarose gel. Primers are listed in Table S4.

Flow Cytometry and Cell Sorting

The following antibodies were used for in vitro cultured cells: CD19 BV421 (Cat# 302234, RRID: AB_11142678), CD33 BV421 (Cat# 303416, RRID: AB_2561690), CD32 PE-Cy7 (Cat# 303213, RRID: AB_2616922), CD14 BV785 (Cat# 301839, RRID: AB_2561366), CD117 BV711 (Cat# 313229, RRID: AB_2566216), CD123 PE-Dazzle (Cat# 306033, RRID: AB_2566449), CD52 PE-Dazzle (Cat# 318917, RRID: AB_2894468), CD163 APC (Cat# 333609, RRID: AB_2291272), CD206 PE (Cat# 321105, RRID: AB_571910), CD11b Alexa Fluor 488 (Cat# 393107, RRID: AB_2734454) (all BioLegend), and CD9 PE (Thermo Fisher Scientific, Cat# 12-0098-42, RRID: AB_10854122). Fixable viability dyes eFluor 506 and eFluor 780 were used (eBioscience, Cat# 65-0866-14, Cat# 65-0865-14).

The following antibodies were used to analyze cells *ex vivo* and sort huCD45$^+$ mCD45$^-$ cells from the BM of NSG mice: CD45 APC (Cat# 304037, RRID: AB_2562049), CD45 BV711 (Cat# 304049, RRID: AB_2563465), murineCD45.1 PE (Cat# 110707, RRID: AB_31349), murine CD45.1 perCP (Cat# 110725, RRID: AB_893347), CD38 PE-Dazzle (Cat# 303537, RRID: AB_2564104), CD15 BV605 (Cat# 323031, RRID: AB_2562131), CD33 BV510 (Cat# 303421, RRID: AB_2565657), CD117 BV711 (Cat# 313229, RRID: AB_2566216), CD9 APC-Fire750 (Cat# 312113, RRID: AB_2728253), CD19 BV421 (Cat# 302234, RRID: AB_11142678), CD14

BV785 (Cat# 301840, RRID: AB_2563425) (all BioLegend). Fixable viability dyes eFluor 506 and eFluor 780 were used (eBioscience, Cat# 65-0866-14, Cat# 65-0865-14).

qRT-PCR

RNA was isolated using the NucleoSpin RNA Kit (Macherey-Nagel, Cat# 740955.50) according to manufacturer’s instructions. For reverse transcription of RNA to cDNA, the RevertAid H Minus First Strand cDNA Synthesis Kit (Thermo Fisher Scientific, Cat# K1631) was used according to manufacturer's instructions. Genes of interest were amplified using Maxima SYBR Green qPCR Master Mix (Thermo Fisher Scientific, Cat# K0222), while the housekeeper 18S rRNA was amplified using Maxima Probe qPCR Master Mix (Thermo Fisher Scientific, Cat# K0261). Primers are listed in Table S5. PCR was performed on a LightCycler 480 Instrument II (Roche Life Science) and CT values were analyzed with the LightCycler 480 Software version 1.5.1. Fold change of *gene* expression was calculated as $2^{-\Delta\Delta CT}$ normalized to 18S rRNA and control cells.

MACE-seq: Data Procession

Raw data were processed with the nfcore/rnaseq pipeline (v3.10.1) against the GRCh38 assembly and v113 of the Ensembl annotations[1]. Stringent quality metrics were applied to ensure high data quality. All samples were included in the analyses. Expression values were imported from the pipeline into R (v4.5.0) and cleaned of lowly-expressed genes (mean expression < 20 normalized reads), leaving about 35 000 genes that were analyzed for differential expression with DESeq2 (v1.48.2)[2]. A gene was deemed differentially expressed if its Benjamini-Hochberg adjusted p-value was <0.05; no fold-change cut-off was applied. Gene set variation analyses are based on MSigDB (v2025.1)[3]. Pathway analysis was carried out using g:profiler[4]. Network topology-based analysis was performed with genes $p_{adj} < 0.2$ using WebGestalt, applying the FunMap HierarchalModules functional database[5, 6].

RNA-seq: Data Procession

Raw data were processed with the nfcore/rnaseq pipeline (v3.19.0) against a custom-built reference genome based on the GRCh38 assembly and v114 of the Ensembl annotations plus the forward and reverse fusion transcripts[1]. Stringent quality metrics were applied to ensure high data quality. All samples were included in the analyses. Expression values were imported from the pipeline into R (v4.5.0) and cleaned of lowly-expressed genes (mean expression < 20 normalized reads), leaving about 19 000 genes that were analyzed for differential expression with DESeq2 (v1.48.2)[2]. A gene was deemed differential if its Benjamini-Hochberg adjusted p-value was <0.05 and its |fold-change| > 0.5. Gene Ontology enrichments were determined with gprofiler2 (v0.2.3)[7], gene set variation analyses were based on MSigDB (v2025.1)[3], and transcription factor activities were predicted with decoupleR (v2.14.0)[8]. Transcription factor enrichment analysis was performed using ChEA3[9].

Isolation of Mononuclear Cells from the Bone Marrow of NSG Mice

Mononuclear cells from the bone marrow were isolated by crushing femur and tibia using mortar and pestle, followed by filtration through a 70 µm mesh. Erythrocytes were lysed by incubation with ACK lysis buffer (155 mM ammonium chloride, 10 mM potassium bicarbonate, 0.1 mM EDTA).

Supplementary Tables

Supplementary Table S1: Target sequences of sgRNAs.

Target	Sequence
AF4	CTGCTGTCGATAGTCCTC
AF9	ATATGGAGAAAGTTGTAG
MLL intron 9	GAGGATCACGAGCCCACA
MLL intron 11	AGCTCCTTATAGATGAAG

Supplementary Table S2: Primers for translocation PCR

Target	Sequence (5´- 3´)
MLL::AF4 Int. 9	Fwd: CCCTGTAAAACAAAAACCAAAAGAA<br>Rev: GCTTTGGCAGGCACTTTCA
AF4::MLL Int. 9	Fwd: GTGAATCCCCTGAACTGAAACC<br>Rev: TGTTCAAAGTGCCTGCATTCTC
MLL::AF4 Int. 11	Fwd: AGTGGACTTTAAGGAGGATTGTGAA<br>Rev: TCAGTTTGGTGAGCTTGGCTT
AF4::MLL Int. 11	Fwd: CCTAGTGAATCCCCTGAACTGAA<br>Rev: GGAAGGGCTCACAACAGACTTG
MLL::AF9 Int. 9	Fwd: CGCCCAAGTATCCCTGTAAAAC<br>Rev: GCTGGCAGGACTGGGTTGT
AF9::MLL Int. 9	Fwd: TGTGGAGGAGAATATATCCTCTAAATCTG<br>Rev: TGCCTGCATTCTCCTGCTTA
MLL::AF9 Int. 11	Fwd: AGTGGACTTTAAGGAGGATTGTGAA<br>Rev: TGGCAGGACTGGGTTGTTC
AF9::MLL Int. 11	Fwd: TTGTGGATCCCAATGATTCAGA<br>Rev: GGAAGGGCTCACAACAGACTTG
KRAS Exon 2*	Fwd:TGTAAAACGACGGCCAGTTATCTGTATCAAAGAATGGTCCTGCAC<br>Rev: CAGGAAACAGCTATGACCGATAGTGTATTAACCTTATGTGTGACATGTTCT
KRAS Exon 3**	Fwd: TGTAAAACGACGGCCAGTTCCTCATGTACTGGTCCCTCATT<br>Rev: CAGGAAACAGCTATGACCGTAAAAGGTGCACTGTAATAATCCAGACT

*Thermo Fisher Scientific, Assay ID: Hs00679698_CE
**Thermo Fisher Scientific, Assay ID: Hs00532900_CE

Supplementary Table S3: Primers for ddPCR

Target	Sequence (5´- 3´)
MLL(9)::AF4	Fwd: ATCACCCTTCCCTGTATTCACT<br>Rev: CCCACATCCTCCGGACATT
MLL(11)::AF4	Fwd: AGCAAAGTTATTGAGAGTGAAAAGA<br>Rev: GGCTTTGGCAGGCACTTTC
MLL(9)::AF9	Fwd: TGACCCCAACATCCTTTAGCA<br>Rev: TGACAGGCCAAATGCTAGGC
MLL(11)::AF9	Fwd: ACAGTGGTCTATTAAAAGGGATGCT<br>Rev: GCCAAGTTGTGAATGCAAAGG
Albumin (*ALB*)[10,11]	Fwd: TGAAACATACGTTCCCAAAGAGTTT<br>Rev: CTCTCCTTCTCAGAAAGTGTGCATAT
Albumin probe[10,11]	GCTGAAACATTCACCTTCCATGCAGA

Supplementary Table S4: Primers for RT-PCR

Target	Sequence (5´- 3´)
MLL::AF4 Int. 9	Fwd: CCCTGTAAAACAAAAACCAAAAGAA<br>Rev: GCTTTGGCAGGCACTTTCA
MLL::AF4 Int. 11	Fwd: AGTGGACTTTAAGGAGGATTGTGAA<br>Rev: TCAGTTTGGTGAGCTTGGCTT
MLL::AF9 Int. 9	Fwd: CGCCCAAGTATCCCTGTAAAAC<br>Rev: GCTGGCAGGACTGGGTTGT
MLL::AF9 Int. 11	Fwd: AGTGGACTTTAAGGAGGATTGTGAA<br>Rev: TGGCAGGACTGGGTTGTTC

Supplementary Table S5: Primers for RT-PCR

Target	Sequence (5´- 3´)
18S rRNA	Fwd: CGGCTACCACATCCAAGGAA<br>Rev: GCTGGAATTACCGCGGCT
HOXA9	Fwd: ATGAGAGCGGCGGAGACA<br>Rev: CGCGCATGAAGCCAGTT
18S rRNA TaqMan probe	[JOE]-TGCTGGCACCAGACTTGCCCTC-[TAM]

Supplementary reference list

1. Ewels PA, Peltzer A, Fillinger S, et al. The nf-core framework for community-curated bioinformatics pipelines. Nat Biotechnol. 2020;38(3):276-278.
2. Love MI, Huber W, Anders S. Moderated estimation of fold change and dispersion for RNA-seq data with DESeq2. Genome Biol. 2014;15(12):550.
3. Subramanian A, Tamayo P, Mootha VK, et al. Gene set enrichment analysis: a knowledge-based approach for interpreting genome-wide expression profiles. Proc Natl Acad Sci U S A. 2005;102(43):15545-15550.
4. Kolberg L, Raudvere U, Kuzmin I, Adler P, Vilo J, Peterson H. g:Profiler-interoperable web service for functional enrichment analysis and gene identifier mapping (2023 update). Nucleic Acids Research. 2023;51(W1):W207-W212.
5. Shi Z, Lei JT, Elizarraras JM, Zhang B. Mapping the functional network of human cancer through machine learning and pan-cancer proteogenomics. Nat Cancer. 2025;6(1):205-222.
6. Zhang B, Kirov S, Snoddy J. WebGestalt: an integrated system for exploring gene sets in various biological contexts. Nucleic Acids Res. 2005;33(Web Server issue):W741-748.
7. Kolberg L, Raudvere U, Kuzmin I, Vilo J, Peterson H. gprofiler2 -- an R package for gene list functional enrichment analysis and namespace conversion toolset g:Profiler. F1000Res. 2020;9(
8. Badia IMP, Velez Santiago J, Braunger J, et al. decoupleR: ensemble of computational methods to infer biological activities from omics data. Bioinform Adv. 2022;2(1):vbac016.
9. Keenan AB, Torre D, Lachmann A, et al. ChEA3: transcription factor enrichment analysis by orthogonal omics integration. Nucleic Acids Res. 2019;47(W1):W212-W224.
10. Pongers-Willemse MJ, Verhagen OJ, Tibbe GJ, et al. Real-time quantitative PCR for the detection of minimal residual disease in acute lymphoblastic leukemia using junctional region specific TaqMan probes. Leukemia. 1998;12(12):2006-2014.
11. Matsuda K, Sakashita K, Taira C, et al. Quantitative assessment of PTPN11 or RAS mutations at the neonatal period and during the clinical course in patients with juvenile myelomonocytic leukaemia. Br J Haematol. 2010;148(4):593-599.
